# Hypersensitivity reactions to metal implants: laboratory options

**DOI:** 10.1186/s12891-016-1342-y

**Published:** 2016-11-23

**Authors:** Anna Maria Carossino, Christian Carulli, Simone Ciuffi, Roberto Carossino, Giorgia Donata Zappoli Thyrion, Roberto Zonefrati, Massimo Innocenti, Maria Luisa Brandi

**Affiliations:** Department of Surgery and Translational Medicine, University of Florence, Viale Pieraccini 6, 50139 Florence, Italy

**Keywords:** Knee arthroplasty, Metal sensitivity, Lymphocyte transformation test, Patch test, Cytokines

## Abstract

**Background:**

All implant compounds undergo an electrochemical process when in contact with biological fluids, as well as mechanical corrosion due to abrasive wear, with production of metal debris that may inhibit repair processes. None of the commonly-used methods can diagnose implant allergies when used singly, therefore a panel of tests should be performed on allergic patients as pre-operative screening, or when a postoperative metal sensitisation is suspected.

**Methods:**

We analysed patients with painful prostheses and subjects prone to allergies using the Patch Test in comparison with the Lymphocyte Transformation Test. Cytokine production was evaluated to identify prognostic markers for early diagnosis of aseptic loosening. Metal debris endocytosis and cytoskeletal rearrangement was visualised by confocal microscopy.

**Results:**

Our results demonstrate that the Lymphocyte Transformation Test can identify patients who have a predisposition to develop allergic reactions and can confirm the diagnosis of hypersensitivity in patients with painful prostheses.

The prevalence of a Th2-cytokine pattern may be used to identify predisposition to the development of allergic diseases, while the selective presence of osteoclastogenic cytokines may be used as predictor of a negative outcome in patients with painful prosthesis.

The hypothesis of the prognostic value of these cytokines as early markers of aseptic loosening is attractive, but its confirmation would require extensive testing.

**Conclusions:**

The Lymphocyte Transformation Test is the most suitable method for testing systemic allergies. We suggest that the combined use of the Patch Test and the Lymphocyte Transformation Test, associated with cytokine detection in selected patients, could provide a useful tool for preventive evaluation of immune reactivity in patients undergoing primary joint replacement surgery, and for clinical monitoring of the possible onset of a metal sensitization in patients with implanted devices.

## Background

Total Knee Arthroplasty (TKA) is one of the most successful orthopaedic procedures for the treatment of osteoarthritis [1, 2]. Particularly high rates of patient satisfaction have been achieved in recent decades thanks to the significant improvement in both surgical technique, implant design, and the characteristics of biomaterials. An increasing number of TKAs have been performed worldwide during recent years as a consequence of the aging population and a higher incidence of secondary osteoarthritis in younger patients [3].

Given the high percentage of hypersensitivity to metals (up to 10%), particularly to nickel, in the general population, and the presence of this particular substance in standard knee implants [4], it may be crucial to verify the patient’s hypersensitivity prior to surgery, in order to prevent reactions. This approach is further justified by the fact that a significant number of failures are expected to occur over the next few decades due to various emerging causes, such as patellar clunk syndrome, periprosthetic fractures, and hypersensitivity to metals [5].

Orthopaedic devices are generally well tolerated, but may sometimes generate corrosion products which cause periprosthetic bone resorption; this can lead to implant looseness and a second surgical procedure may be required to fix or replace the failed implant [6, 7].

The presence of small amounts of debris that can be removed through catabolic processes is consistent with biological tolerance of implants [8, 9], while high concentrations of free metal ions may be accumulated in the surrounding tissue, or carried through the bloodstream to distant organs [10, 11]. Metal particles bind to serum proteins to form hapten-like complexes that may be identified by the immune system as antigens, and can activate local or systemic inflammatory reactions by recruiting macrophages, fibroblasts, lymphocytes, and osteoclasts, which induce proinflammatory and osteoclastogenic cytokine release [12]. This response, classified as a type-IV delayed hypersensitivity reaction, is mediated by antigen-presenting cells and T lymphocytes, and can occur either in the postoperative period or months and even years later [7, 13].

T cell activation produces a self-perpetuating loop whereby macrophages are recruited and activated, these in their turn present a class II major histocompatibility complex (MHC II) which can activate other sensitised T cells, and so on. Soluble ions and metal particles may induce monocyte/macrophage activation that stimulates the inflammatory response by various mechanisms. Particles can be internalised by phagocytosis, which is traditionally associated with the expression of MHC II on macrophages, while soluble ions can penetrate cells via passive or active transport.

Several studies have demonstrated that cell response may be affected by the size, shape, quantity and composition of the debris, and have suggested a possible correlation between the presence of corrosion products and the symptoms of localized or systemic allergic dermatitis [14, 15]. Nevertheless, there is no evidence of a direct link, since immune response changes in relation to the general state of health, genetic susceptibility, and individual hypersensitivity [16].

The incidence of serious adverse reactions requiring implant removal is quite low, but the percentage of patients with postoperative local inflammatory symptoms, persistent pain, and poorly- functioning implants is not negligible, and demands thorough evaluation. Because the immune-related mechanisms of metal allergy development are not well understood, and several factors may contribute to overaggressive responses, this phenomenon may be underrated in the evaluation of persistent post-surgical pain.

To date, no in vivo and in vitro methods have been specifically assessed for their efficiency in testing allergies caused by implants. Patch testing (PT) tends to be used as the standard test because of its cost-effectiveness and technical simplicity, but its specific diagnostic usefulness is controversial. In fact, there is a remarkable difference between the immunological reaction deriving from antigen-presenting cells located in the skin layers and the systemic response to metal debris, which is mediated by macrophages and dendritic cells located in the periprosthetic area. Moreover, screening is not recommended because hapten exposure may itself induce sensitization. In addition, visual scoring of skin reactions is conditioned by the physician’s experience and may be influenced by medications, the quality of the substances, and the time of reading [17, 18].

More specific laboratory methods, based on lymphocyte proliferation and measurement of cytokine release, have been proposed: the Lymphocyte Transformation Test (LTT), which evaluates the proliferative response of activated T lymphocytes, is based on evidence that allergic individuals produce memory T cells which are able to be activated by antigen exposure. Moreover, the detection of specific cytokines secreted as the result of an immune response indicates the qualitative and quantitative involvement of different cell types [19, 20].

Generally, the diagnosis of metal allergy in patients who have undergone a second surgical procedure due to the negative outcome of the first one is made by exclusion criteria: lack of evidence of infections, non-union, and mechanical failure. When an aseptic loosening is confirmed, revision surgery is the only option. In addition to the patient’s suffering, the complexity and time-consuming aspects of the revision procedures, as well as the related social and economic costs must all also be taken into account. It is thus useful, in order to choose the correct implant, to identify which metal is inducing the allergic reactions, [21, 22] both in allergic subjects and in patients with persistent post-surgical pain. TKA offers a unique model of study of such condition because it is the only procedures that may be performed using actual fully non-allergic implants.

### Purpose of this study

We selected a group of patients with a clinical history of metal allergies who had undergone primary Total Knee Arthroplasty (pTKA), and a second group of patients with painful prostheses. Each patient was compared to a group of control subjects.

The aim of the study was to assess the ability of LTT and PT to discriminate between skin and systemic reactions. Cytokine production was evaluated to identify prognostic markers for early diagnosis of aseptic loosening. Finally, to assess metal endocytosis and cytoskeletal rearrangement, cell interaction with metal particles was visualised by confocal microscopy.

## Methods

### Patients

Thirty potentially allergic patients, divided into two groups, were studied. Demographic and clinical data were collected using a questionnaire. Group 1 included eight patients scheduled for pTKA, who had a documented clinical history of metal allergy and hypersensitivity reactions (eczematous rashes, rhinitis, asthma). Group 2 was composed of twenty-two patients with a painful TKA. These were evaluated for the nature, location, onset and duration of their pain, at least 6 months postoperatively, and their pain was classified on the basis of a Visual Analogical Scale ≥7. All signs of the common causes of failure (infection, instability) were ruled out. Eleven of the patients referred metal allergies (Group 2A), while the other eleven did not refer any allergies and had no clinical signs of sensitisation (Group 2B).

All patients were studied by medical history, blood tests for infection indices (hemochrome, CRP, ESR) and radiological evaluation (anterior-posterior, lateral and patellar views, long-standing radiograms, CT scan for rotational evaluation of components). Functional limitation was measured using a Range of Motion (ROM) evaluation, and by the Knee Society Score (KSS) and the Western Ontario and McMaster Universities Osteoarthritis Index (WOMAC).

Controls (Group 3) included nine volunteers unaffected by skin disorders or immunological, metabolic, or chronic diseases, and without any previous known contact with metal implants.

### Patch test

All subjects were tested according to the guidelines suggested by the Società Italiana di Dermatologia Allergologica Professionale e Ambientale (SIDAPA) based on international guidelines [23, 24].

Metal allergy was tested by using the following haptens: Cobalt Chloride 1%, Nickel Sulphate 5%, Potassium Dichromate 0.5%, and Chromium III 2%. (F.I.R.M.A SpA, Florence, Italy). Vaseline, used as vehicle for patch test, was assayed as a negative control.

A drop of each hapten was smeared on Haye’s chamber test, which was applied to areas of the left side of the patient’s upper back that were free of erythema and dermatitis.

The reading was performed after 48 and 72 h, and results were recorded based on the second reading. According to the recommendation from the International Contact Dermatitis Research Group, allergic responses from 1+ to 3+ were interpreted as a positive reaction, and were scored as: 1+ (week non-vesicular erythema with edema and infiltration), 2+ (moderate homogeneous redness, with edema, infiltration and vesicles), and 3+ (strong homogeneous redness, infiltration and bullous reaction). A negative reading (0) or a doubtful reaction (+?, only erythema without infiltration) was interpreted as a negative response [25].

### Lymphocyte transformation test

Samples were collected after informed consent and before PT, to prevent sensitisation. Peripheral Blood Mononuclear Cells (PBMC) were isolated with Lymphocyte Separation Medium and resuspended in RPMI-1640 containing 10% Fetal Bovine Serum. Non-toxic concentrations of challenge metals (0,1 mM; 0,01 mM) were selected by a dose response curve. 2 × 10^5^ cells/well were seeded in triplicate in 96-well plates with or without Chromium (III) chloride (CrCl_3_), Chromium powder (Cr), Nickel (II) chloride (NiCl_2_), Nickel nanopowder (Ni), Cobalt powder (Co), Titanium powder (Ti), and Molybdenum nanopowder (Mo). Ni and Cr, the main sensitiser metals, were analyzed both in soluble and particulate form. Phytohemagglutinin (PHA 0,01 mg/ml) was used as control. After 5 days, cells were pulsed overnight with ^3^H-thymidine (1 μCi/well) and proliferation was assessed by scintillation counting. Results were expressed as Stimulation Index (SI = mean cpm-treated /mean cpm-untreated cultures). Culture media and supplements were purchased from Biowhittaker, (Lonza, Treviglio, Italy), chemicals from Sigma Aldrich (Milan, Italy).

### Luminex cytokine assays

Cytokine production was evaluated in eight Group 1 patients, four Group 2A patients (all positive to LTT) and five Controls. Supernatants from metal-challenge PBMC were collected on day 5 and stored at −80 °C. Luminex multiplex array (Bio-Rad Laboratories, Hercules, CA, USA) was used to quantify: IL-1β, IL-1ra, IL-2, IL-4, IL-5, IL-6, IL-7, IL-8, IL-9, IL-10, IL-12, IL-13, IL-15, IL-17, eotaxin, bFGF, G-CSF, GM-CSF, IFN-γ, IP-10, MCP-1, MIP-1α, MIP-1β, PDGF-BB, RANTES, TNF-α, VEGF. Samples were analysed in duplicate using the reagents and the protocol supplied in the kit.

Out-of-range values (above or below detection limits) were rated as the highest and the lowest detectable concentrations. Cytokine production was expressed as Stimulation Index (SI = value of stimulated cultures/ value of unstimulated cultures).

### Phase-contrast and laser scan confocal microscopy (LSCM)

PBMC (2 × 10^4^ cells/well) were cultured in LabTek chamber-slides (Thermo Scientific-NUNC, Milan, Italy) for 5 days with or without 0.1 mM and 0.01 mM of selected metals. Reagents were purchased from Sigma Aldrich Milan, Italy. Cells were fixed with 4% paraformaldehyde, permeabilised in 0.2% Triton X-100, and non-specific binding sites blocked using 2% Bovine Serum Albumin. To evaluate the cytoskeletal rearrangement of F-actin filaments, samples were stained with Phalloidin TRITC-conjugate. Nuclei were counterstained with Hoechst 33258. Samples were mounted in polyvinyl alcohol mounting medium with anti-fading DABCO and examined under an Axiovert 200 M inverted LSM510 (Carl Zeiss, Oberkochen, Germany). Quantitative assessment of cells with internalized metals was carried out by counting a total number of 200 cells/well, per metal treatment. Quantification of positive cells was expressed as the percentage fraction of total cell numbers counted for each metal.

### Statistical analysis

The mean values of SI in allergic patients labelled for TKA and in patients with painful prostheses were compared to those of control subjects. The differences among the groups were evaluated using the ANOVA procedure (STATA statistical package, Stata Corp. 2009 - Stata Statistical Software: Release 10. College Station, TX: Stata Corp LP), and the REGRESS post estimation command was used to calculate the *p*-values of the differences between each group of patients (allergic, painful prosthesis) and control subjects.

Previous literature was analysed to perform a power calculation. Since several studies did not report effect size statistics, a raw estimate of an effect size was achieved using nonparametric statistics reported in conditions which were as similar as possible to ours [26].

The agreement between PT and LTT was estimated in absolute percentage and by means of the Kappa Statistic (0–0,20 = poor; 0,21-0,40 = fair; 0,41-0,60 = moderate; 0,61-0,80 = good; 0,80-1 = very good), which takes chance agreement into account [27].

## Results

Considering the relatively small sample size, we tried to perform a power calculation by analysing previous literature. Several studies did not report effect size statistics and often did not provide enough data to compute them. The raw estimate is between .49 and .64. In our case, with this effect size, comparing the 4 groups with ANOVA, the power is between 71 and 91%.

### Patch test and lymphocyte reactivity

**S**ince none of the laboratory tests are able to provide certain diagnoses of allergy, we tried to correlate PT with the LTT. PT was evaluated according to the clinical scoring criteria of the International Contact Dermatitis Research Group [25].

The LTT was considered positive for SI > 2. The enhanced proliferation was explained as the expression of a metal-specific lymphocyte response. All control subjects were found to be negative to PT as well as to the LTT. The demographic and clinical characteristics of the 30 patients, and their individual reactivity to PT and the LTT, are summarised in Table 1. The two tests matched in 60% of cases. A complete correspondence (PT-LTT- or PT + LTT+) for the same metals was achieved in 37% of tests. Contradictory results (PT + LTT-) were obtained in 13% of cases, while (PT-LTT+) in 17%. Doubtful PT (10%) were ruled out by the LTT (7% negative and 3% positive responses). The PT and LTT results obtained for each group of patients are detailed in Table 2.

**Table 1:** Overview of demographic and clinical characteristics, and results of PT and LTT

Group 1	Gen	Dental fillings	Familiarity for allergy	Professional exposure	Known allergies	Jewelry allergy	Cosmetic allergy	PT reactivity	LTT response
1	F	Yes	None	None	Ni	Yes	None	++++Ni	++++Ni,+Cr,
2	F	Yes	Yes	None	Ni	Yes	Yes	++Ni	+++Ni,+Cr,+Ti
3	F	Yes	None	None	Ni	Yes	None	++Ni,+Co	+Ni
4	F	Yes	Yes	None	Ni	Yes	None	+++Ni	+Ni,+Cr
5	F	Yes	None	None	Ni,Cr	Yes	None	+Ni	Neg
6	F	Yes	None	Yes	Ni	Yes	Yes	++Ni	Neg
7	F	Yes	None	None	None	Yes	Yes	+Ni	+Ni
8	F	Yes	None	None	None	Yes	Yes	+/−Ni	+Ni
Group 2A									
1	F	None	None	None	Ni	None	None	++Ni	+Ni
2	F	Yes	None	None	None	None	None	++Co	+Ni,+Cr,+Mo
3	F	None	None	Yes	Ni	Yes	None	++Ni	++Ni,+Cr
4	F	None	None	Yes	Ni	Yes	None	++Ni	+ Ni
5	F	Yes	None	None	Ni	Yes	None	++Ni	+ Ni
6	F	Yes	None	None	Ni	Yes	None	++Ni	++Ni
7	F	Yes	None	Yes	Ni	Yes	Yes	Neg	+Ni
8	F	Yes	None	None	Ni	Yes	Yes	Neg	+Ni
9	M	Yes	None	Yes	Ni	None	Yes	Neg	+Ni,+Cr,+Co,+Ti
10	F	None	None	None	None	Yes	None	Neg	+Ni,+Cr
11	M	None	None	None	None	None	None	Neg	+Ni
Group 2B									
12	F	Yes	Yes	None	None	Yes	Yes	Neg	Neg
13	F	Yes	None	None	None	None	None	Neg	Neg
14	F	Yes	None	None	None	None	None	Neg	Neg
15	M	Yes	None	None	None	None	None	Neg	Neg
16	F	Yes	None	None	None	Yes	Yes	Neg	Neg
17	F	Yes	None	None	None	Yes	None	Neg	Neg
18	F	None	None	None	None	None	None	Neg	Neg
19	F	Yes	None	Yes	None	Yes	None	+Cr	Neg
20	M	Yes	None	None	None	None	None	+Ni,+Co	Neg
21	F	None	None	None	None	None	None	+/−Co	Neg
22	M	Yes	Yes	None	None	None	None	+/− Cr	Neg

**Table 2:** Number and percentage of stimulatory responses revealed by PT and LTT

Group 1					
	Number of cases	%		Number of cases	%
PT+	7/8	87,5	LTT+	6/8	75
PT-	----		LTT-	2/8	25
PT+/-	1/8	12,5			
Group 2A					
	Number of cases	%		Number of cases	%
PT+	6/11	54,5	LTT+	11/11	100
PT-	5/11	45,5	LTT-	---	---
Group 2B					
	Number of cases	%		Number of cases	%
PT+	2/11	18	LTT+	---	---
PT-	7/11	64	LTT-	11/11	100
PT+/-	2/11	18			

The observed agreement between the two tests calculated by the Kappa Statistic, not considering PT as a gold standard, was 76.6% for all metals tested, with a Kappa value of 0.3 (fairly good agreement). Similar results were obtained by comparing the two tests for each metal.

**Fig. 1:**
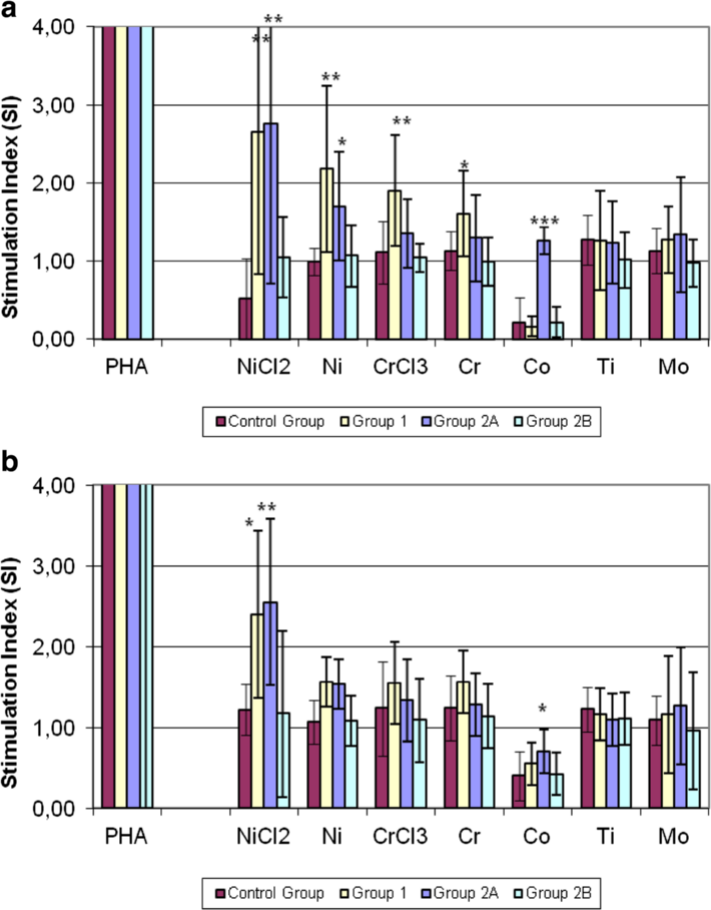
**a**, **b** Lymphocyte Transformation Test Response. The effect of various metals and PHA on the proliferation rate of patients’ and controls’ lymphocytes: mean lymphocyte response (SI) of each of the three patient groups compared to controls. Group 1 = patients scheduled for pTKA, labelling for a clinical history of metal allergy; Group 2A = TKA patients with pain and clinical signs of metal allergies; Group 2B = TKA patients with pain and no clinical signs of metal allergies; Group 3 = Control subjects. Metal concentrations: (**a**) = 0.1 mM; (**b**) = 0,01 mM. Asterisks indicate: (*) *p* < 0.05; (**) *p* < 0.005; (***) *p* < 0.0001

Figure 1a,b shows the effect of sensitiser metals on lymphocyte response. Mean SI values for each of the 3 patient groups were compared to controls. At the concentration of 0.1 mM, (Fig. 1a), NiCl_2_ produced the highest degree of proliferative response, with a more than fivefold increase in Group 1 and Group 2A (5.1 and 5.3 respectively), and a twofold increase in Group 2B. Statistically significant difference was reached in Group 1 (*p* = 0.004) and Group 2A (*p* = 0.001). Moreover, Ni increased proliferative response by about twofold in Group 1 and Group 2A (2.2 and 1.7 respectively), with a statistically significant difference in Group 1 (*p* = 0.001), and Group 2A (*p* = 0.01). Group I patients showed a statistically significant proliferative response in the presence of CrCl_3_ (1.7; *p* = 0.001) and Cr (1.4; *p* = 0.03), while Group 2A patients showed a high proliferative response, about a sixfold increase, in the presence of Co (5.7; *p* < 0.0001).

At a concentration of 0.01 mM (Fig. 1b), CrCl_3_ Cr and Ni did not produce statistically significant responses. In the presence of NiCl_2,_ Group 1 and Group 2A showed about a twofold increase in proliferative response (2; *p* = 0.01 and 2,1; *p* = 0.003). Co increased proliferative response to a lesser extent (1.7; *p* = 0.01). Ti and Mo did not produce statistically significant proliferative effects, remaining constantly below the threshold value of SI < 2. No significant differences were detected between the two concentrations of sensitiser metals.

### Luminex cytokine assays

**Fig. 2:**
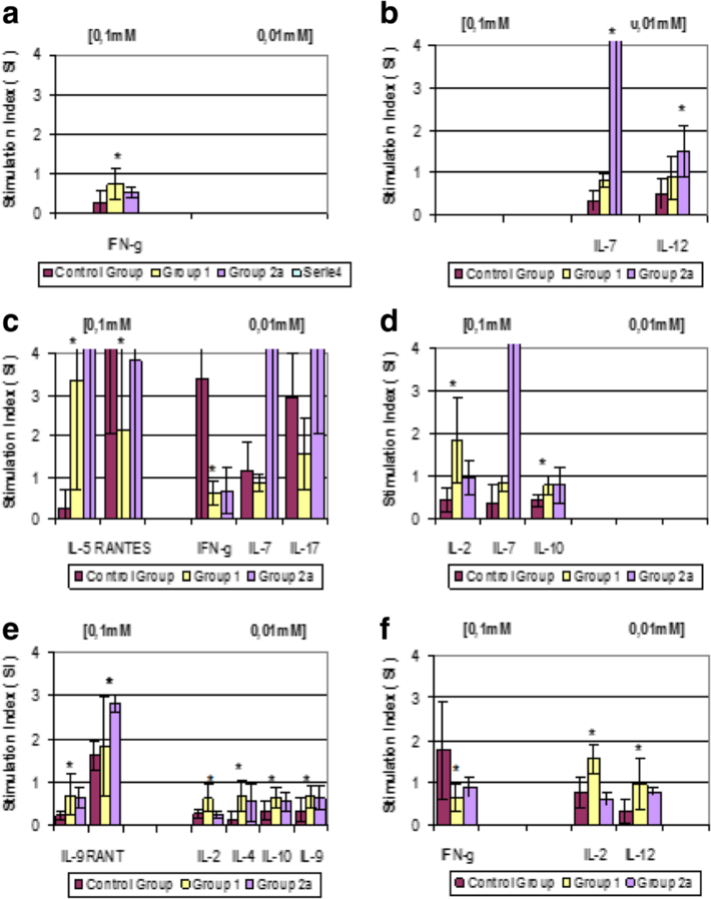
**a**-**f** Cytokine production in response to metals. Cytokine production in response to metals (0.1 mM and 0.01 mM) in the two patient groups compared to controls. Group 1 = patients scheduled for pTKA, labelling for a clinical history of metal allergy; Group 2A = TKA patients with pain and clinical signs of metal allergies; Group 2B = TKA patients with pain and no clinical signs of metal allergies; Group 3 = Control subjects. Metals: (**a**) = Chromium chloride; (**b**) = Chromium; (**c**) = Nickel chloride; (**d**) = Nickel; (**e**) = Cobalt; (**f**) = Molybdenum. (SI = mean of Stimulation Index). Asterisks indicate: (*) *p* < 0.05; (**) *p* < 0.005

Although a wide range and sometimes large amounts of cytokines were produced, only some cytokines reached statistically significant levels, owing to the low number of subjects analysed (Fig. 2a-f).

The ratio of stimulated to unstimulated cultures in cytokine production was expressed as SI and averaged. Statistically significant differences were obtained comparing each of the two patient groups with controls.

Ti did not activate cytokine production in any of the analysed cultures.

Cells from allergic patients with no prosthesis revealed a high reactivity to Ni and a mild reactivity to Mo, Co and Cr, resulting in the prevalent induction of cytokines which were indicative of T-cell activation (IL-5, IL-2, IFNγ) and cytokines associated with monocyte/macrophage activation (IL-10).

Cells from allergic patients with painful prostheses revealed a high reactivity to Cr with statistically significant production of IL-7 and IL-12. Increased production of IL-5, RANTES, IL-7 and IL-17 was observed under Ni stimulation. Comparison between patients with painful prostheses and allergic patients showed statistically significant differences (*p* = 0,027) only for IL-17. Co induced a statistically significant production of RANTES.

### Phase-contrast and laser scan confocal microscopy

Cell interaction with metal particles was visualised by confocal microscopy, to evaluate cytoskeletal rearrangement and metal endocytosis.

Metal debris within cell cytoplasm can be observed as non-fluorescent dark areas of different shapes and sizes, which are not detectable in unstimulated cultures.

**Fig. 3:**
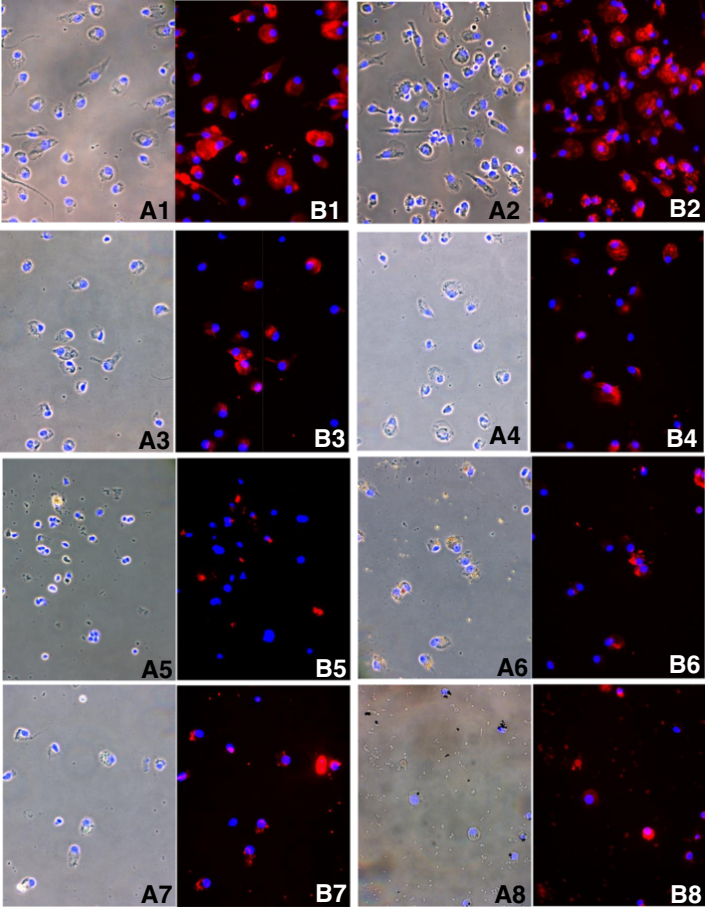
Phase-contrast microscopy. Representative images of cells stimulated by different metals (0.1 mM). Panels **a** and **b** represent phase contrast (*A1*→*A8*) and fluorescent images (*B1*→*B8*), overlapped with counterstained nuclei. (*A1-B1*: untreated cells; *A2-B2*: Ti; *A3-B3*: Co; *A4-B4*: Mo; *A5-B5*: NiCl_2_; *A6-B6*: Ni; *A7-B7*: CrCl_3_; *A8-B8*: Cr-treated samples. (Phalloidin TRITC-conjugate and Hoechst 33258). (1-3; 5-7 = 40× original magnification), (4; 8 = 63× original magnification)

Generally, Ti, Mo and Co seemed to have non-toxic effects resulting in well-preserved cell morphology, while marked cytoskeletal alterations were found following exposure to Ni and Cr. Representative images of cells stimulated by different metals (0.1 mM) are shown in Fig. 3. Panels A and B represent phase contrast (A1→A8) and fluorescent images (B1→B8) with overlapping counterstained nuclei.

No signs of cellular distress were found in either untreated cells (A1, B1) and Ti-stimulated cells (A2, B2). Some minor cytoskeletal modifications were seen in Co (A3, B3) and Mo (A4, B4) -treated cells, which in some cases were similar to controls. Conversely, destruction of cytoskeletal components was evident in NiCl_2_ (A5, B5) and Ni (A6, B6) -treated samples. Moreover, CrCl_3_ (A7, B7) and Cr (A8, B8) caused multiple defects in the cytoskeleton, and the cytoplasm appeared markedly damaged or partially destroyed.

**Fig. 4:**
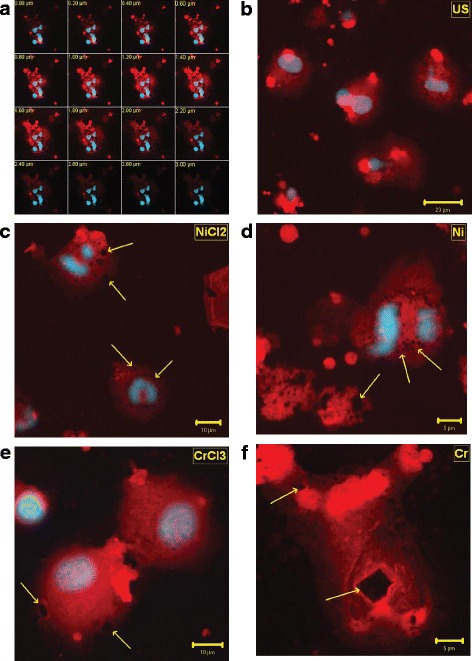
**a**-**f** Confocal Microscopy. Representative images of z-stacks optical sections (**a**), untreated cells (**b**), NiCl_2_ (**c**), Ni (**d**), CrCl_3_ (**e**), and Cr (**f**) -stimulated cells. (Phalloidin TRITC-conjugate, Hoechst 33258). (Phalloidin TRITC-conjugate, Hoechst 33258). b = 40x, c,e = 63x, d,f = 100x original magnification

Confocal optical sections (z-stacks) confirmed the internalisation of metal particles, which appeared in sequential images as multiple dark areas inside the cytoplasm (Fig. 4a). Similar results were obtained in all samples. Representative images are shown in Fig. 4b-f.

Untreated cells (Fig. 4b) showed round or oval shapes, with central, slightly enlarged nuclei. No dark areas were observed inside the cytoplasm.

Ti, Mo and Co-stimulated cells appeared similar to controls (data not shown).

In the attempt to obtain a quantitative evaluation, the percentages of cells containing metal debris were calculated.

Generally, high percentages of damaged cells were found in Ni and Cr-stimulated cells, while low percentages of positive cells were found in Ti, Mo and Co-stimulated cells, except for the few patients with specific Ti, Mo or Co-sensitivity.

Similar behavior was observed for the two concentrations of sensitizer metals, but statistical significance was not reached, owing to the lack of uniformity in cell counts among the patients. In fact, at the concentration of 0.1 mM, the percentage of cells containing even a little amount of metals, varies from 2 to 17% for Ti, from 2 to 18% for Mo, and from 4 to 22% for Co.

On the other hand, NiCl_2_ and Ni -treated cells (Fig. 4c,d) showed clusters of metal debris surrounding the nucleus, which was still completely enclosed in the cytoplasm, as well as internalised aggregated particles in fragmented cells. The percentage of damaged cells varied from 70 to 82% for NiCl_2_ and from 47 to 55% for Ni. Multiple dark holes due to phagocytosed particles were also found in CrCl_3_ and Cr-treated cells (Fig. 4e,f), with percentage of damaged cells varied from 40 to 48% and from 40 to 44%, respectively.

Morphological changes, such as disruption of cell membrane and disappearance of nuclei, indicated severe cell injury.

### Clinical outcome

All Group 1 patients with hypersensitivity diagnosed by PT and LTT underwent TKA with a non-allergic implant (Genesis II, Smith & Nephew, Memphis, TN), characterised by an Oxidised Zirconium femoral component, and all PolyEthylene (PE) tibial and patellar components.

At the 12-month post-surgical evaluation, no complications were observed, and all patients referred a good outcome.

On the basis of clinical evidence, seven of the Group 2A patients with hypersensitivity, diagnosed by LTT alone or PT plus LTT, underwent revision arthroplasty using a Nickel-free implant with an Oxidized Zirconium femoral component (Legion OxZr/PE/Ti, Smith & Nephew, Warsaw, IN) or a Zirconium Nitride multicoating implant (E-motion multicoating NiZr/PE, BBraun, Melsungen AG). The other four patients were monitored by a strict follow-up and treated by pain-controlling drugs, owing to the individual risk of possible complications due to concomitant disease. They underwent revision surgery using the Nickel-free implant (Legion OxZr/PE/Ti, Smith & Nephew, Warsaw, IN). At the 12-month postoperative follow-up, no complications were observed, and patients referred relief from symptoms and a satisfactory functional recovery. Two subjects referred clinical improvement, but with persistent swelling and pain. They are still under observation in order to understand the reason for this partial recovery.

Group 2B patients, who all showed negative LTT but different responses to PT, were considered as not allergic to metals. In three out of eleven patients who had undergone a second surgical procedure with a Nickel-free implant (Legion OxZr/PE/Ti, Smith & Nephew, Warsaw, IN), the pain had disappeared. The other patients, kept under clinical observation and treated with oral analgesics and periodical steroid drug administration, referred persistent symptoms and a variable joint function.

## Discussion

Several studies were performed to analyse the possible relationship between metal hypersensitivity and painful or poorly-functioning prostheses. The individual conditions which may favour the onset of post-surgical complications are as yet unknown. None of the clinical or laboratory analyses prove that these complications may be caused by metal particles released from implants or by pre-existing metal sensitivity. Nevertheless, in vitro testing for metal allergies is recommended for patients undergoing arthroplasty who have known hypersensitivity reactions.

In our study, all laboratory results were considered in the decision-making process, and treatment options were tailored according to the patient’s needs. Even so, some patients with painful prostheses referred only a partial recovery after revision surgery. As discussed elsewhere, the persistence of post-surgical pain is not clearly defined [28, 29], but Macrae reports that surgery itself is the second most common cause of this problem [30]. A high failure rate in a revision series (about 80%) has been reported in cases of undefined pain origin [31]. The persistence of pain may be a consequence of underlying conditions, or of surgical complications. Wylde et al. assess that 44% of patients undergoing TKA suffer from persistent post-surgical pain of varying severity [32] and revision surgery may also lead to uncertain clinical results.

These data highlight the need to have as much information as possible concerning the nature of pain. Moreover, there is an urgent need to develop in vitro tests to achieve better understanding of this problem. Current laboratory methods, used individually, failed to provide a sure diagnosis. To offset the limitations of each method, a combined approach evaluating both PT and LTT was used to detect systemic metal reactivity. When a diagnostic test is unable to specifically identify a clinical condition, it must be double-checked with another test to confirm a preliminary diagnosis or clarify contradictory responses.

PT may not reflect the immunological response at the implant site. A positive PT indicates an allergen-specific cutaneous reaction, but does not clearly signify the development of metal sensitivity. PT itself may also induce sensitization. Moreover, strong reactions indicate true positivity, but it is very difficult to interpret a doubtful result [33]. Nevertheless, it must always be considered within the overall clinical context, or be supported by other findings. Only more in-depth analysis can rule out or confirm allergic response and identify the specific sensitiser metal.

LTT may be more useful than PT in allergic patients, ruling out a direct contact with allergens as well as doubtful PT results. Therefore, considering that PT alone is inadequate for formulating a diagnosis or for deciding on revision surgery, we explored the potential of LTT as a confirmatory test. The limited agreement between PT and LTT shown by the Kappa coefficient is probably due to the small cohorts analysed.

Sometimes PT reactivity was not confirmed by LTT. False-positive or negative reactions in patients affected with contact dermatitis have been documented by Brasch et al. and Sarma [34, 35]. In our experimental conditions, 87% of Group 1 patients tested positive to PT. LTT confirmed and sometimes expanded the diagnosis of metal reactivity in 75% of cases. Negative LTT results were explained as skin-specific sensitivity.

All Group 2A patients were found positive to LTT but only 55% to PT. This may be indicative of a systemic sensitisation, but it means that PT failed to identify sensitised subjects in 45% of cases. At the same time, all Group 2B patients were found negative to LTT. Again, a clearly negative response to PT was detected in only 64% of cases. Since comparison with other methods is lacking, this constitutes a serious limit to the clinical decision process.

These results supported our working hypothesis, whereby LTT is a more suitable method for testing systemic allergies and differentiating dermal from implant-induced hypersensitivity reactions. However, the lack of overlap suggests that these tests are complementary but not equivalent. In fact, they recognise different biological mechanisms which are only partially correlated. This is borne out by the comparison of mean LTT SI values among the 4 groups. In fact, allergic subjects affected by knee arthritis and hypersensitivity to metals diagnosed by PT and LTT had comparable SI values to those of patients with painful arthroplasty, while patients with painful arthroplasty but no clinical signs of sensitisation showed similar results to controls. No significant differences were detected between the two concentrations of sensitiser metals.

LTT response to NiCl_2_ and Ni was significantly higher in Group 1 and Group 2A patients. Statistically significant effects were also determined by CrCl_3_ and Cr in Group 1 patients, despite the fact that all values constantly remained below the SI threshold value.

Interestingly, Co produced a remarkable degree of proliferative response only in Group 2A patients. The high statistical significance of these findings is consistent with the clinical characteristics of these patients. Moreover, a dose–response relation between the two metal concentrations was also observed. No statistically significant differences in Ti and Mo responses were detected among the four groups.

These findings showed that positive LTT response may indicate patients who have a predisposition to develop pathological reactions to implanted devices, and confirm the suspicion of metal allergy in patients with painful prostheses. Thus in case of limited resources, we suggest that LTT should be preferred to PT.

Cytokine assay may be reserved for patients undergoing revision surgery.

Regarding cytokine production, in allergic patients with no prosthesis Ni caused a statistically significant increase in IL-5, which is considered an allergen-specific cytokine. Czarnobilska et al. showed that Nickel-induced IL-5 production is related to the intensity of PT response and might be a useful marker to distinguish between allergic and non-allergic subjects [36].

IL-5 was also reported to be secreted in vitro by nickel-stimulated peripheral blood mononuclear cells in Ni-allergic patients. All these results indicate that type 2 response might contribute to the immunopathogenesis of contact hypersensitivity [37–39].

Also a statistically significant increase in IL-2 was evidenced, in agreement with previous reports that indicate IL-2 as an early occurring type-1cytokine in patients with Ni allergic contact dermatitis [40].

A statistically significant production of RANTES, which may recruit leukocytes at the site of inflammation, was found. Other cytokines potentially involved in allergic responses (IL-4, IL-9) were detected. IL-7 regulates Th1/Th2 cytokine production, while IL-2 and IL-10 can play immune-suppressive or stimulatory roles. An increase in IL-2 and a decrease in IFN-γ may induce a switch of the T helper lymphocytes. The up-regulation of Th2 and Th9 cells and the down-regulation of Th1 cells reveal a typical allergic pattern in this group of patients. All these data are coherent with the significantly higher proliferative response to Ni, found by the LTT test, in this group.

In allergic patients with painful prostheses the high reactivity to Co enhanced the production of RANTES, which may recruit leukocytes at the inflammation site. Other metals induced the production of cytokines associated with monocyte-macrophage activation, which plays a role in the regulation of type1/type2 cytokine balance (IL-12). Elevated IL-12 levels have been reported by Inomoto et al. in the pseudosynovial fluid of patients with aseptic loosening of hip prostheses [41].

A statistically significant increase was observed in IL-7 production under Cr stimulation and IL-17 production under Ni stimulation. These osteogenic cytokines can stimulate RANKL production. As is known, the RANK-RANKL system stimulates osteoclast activation, increasing bone resorption [42, 43]. Increased levels of these cytokines are consistent with the clinical characteristics of the patients analysed, and can be related to implant failure. Also in this group of patients the increased response to Ni and Co observed by LTT are coherent with the production of osteoclastogenic cytokines which are specific for these patients.

This is in accordance with Summer B. et al. that showed that patients with complicated total joint arthroplasty and concomitant Ni patch test reactivity had a predominant IL-17 response. This suggests that a potentially increased risk of complications following prosthesis implantation might be based on the evaluation of IL-17 [4].

Regarding the cytokine profile, the observed data are insufficient to support the need for routine cytokine assay as a preliminary test. In our experimental conditions, a different response was found in the two patient groups. All metals induced a mixed Th1 and Th2-type cytokine production. Despite the small number of cases, the prevalence of a Th2-cytokine pattern may be used to identify predisposition to the development of allergic diseases. At the same time, the selective presence of osteoclastogenic cytokines as IL-17 and IL-7 and the evidence of RANTES and IL-12, may be used as predictors of a negative outcome in patients with painful prosthesis.

However, because of the high inter-individual and intra-individual variability of cytokine expression, a population study needs to be performed to ascribe prognostic values to specific cytokines.

An alternative approach is represented by molecular analysis of cytokine pattern together with histological evaluation of the periimplant tissue, in patients undergoing revision surgery.

Hercus B. et al. analyzed mRNA expression of TH1 (IFN- γ, TNF- β, IL-2, IL-12) and TH2 (IL-4, IL-5, IL-6, IL-10) cytokines present at the bone-implant interface of aseptically loosened joints, and demonstrated a predominance of TH1 over TH2 response [44].

Recently, Thomas suggested a combined approach to evaluate a possible link between a specific cytokine expression pattern and periimplant tissue analysis, in patients with TKA failure. An high expression of IFN- γ and IL-2 was evidenced with semiquantitative real-time RT-PCR [26, 45].

Regarding histological analysis of periprosthetic tissues, past investigations evidenced the presence of metallic aggregates in the perilesional area, as well as perivascular infiltrations of T-cells and macrophages in the tissue sections [46, 47].

Davies A.P. et al. described arthroplasty failure in association with the histological evidence of periprosthetic lymphocytic infiltration and macrophage containing metal debris [48].

Witzleb W.C. et al. reported that the presence of diffuse and perivascular lymphocytic infiltration in periprosthetic tissue may be considered to be a characteristic histological pattern of tissue reactions to metal particles [49].

Other Authors analysed some histological parameters and cytokine levels in tissue and synovial fluids from patients undergoing primary total hip/knee replacement and from patients requiring revision for aseptic loosening. Statistically significant increases in cytokine levels and in macrophage infiltrate were found in samples from patients with aseptic loosening, compared to patients undergoing primary surgery [50, 51].

For histopathological classification of the hypersensitivity reactions in periprosthetic tissue, a standardized consensus classification was established [52].

Nevertheless, microscopy approach remains a qualitative method, generally referred to limited groups of cells. Other limitations have arisen from the heterogeneity of implant devices, source and location of tissue samples, patient characteristics and because of the absence of proper control groups.

All of these investigations concern patients requiring hip or knee revision surgery for ascertain aseptic loosening of prosthesis.

Our study design aimed to identify common laboratory methods for the preventive evaluation of potentially allergic patients and for the early diagnosis of aseptic loosening in patients whit painful prosthesis.

Phase-contrast microscopy was preliminarily used on PBMC to reveal changes in cytoskeletal structure, and Laser Scan Confocal analysis was applied to visualise solid particles internalisation and their effects on cytoskeleton rearrangement.

Generally speaking, phase-contrast microscopic evaluation revealed that Ti, Mo and Co had non-toxic effects resulting in well-preserved cell morphology.

Conversely, after exposure to Ni and Cr, the number of cells appeared to be strongly reduced and marked cytoskeletal alterations were revealed.

Also LSCM observation confirmed the relatively low toxicity of Ti, Mo and Co, while under Ni and Cr stimulation, an high percentage of cells with a considerable amount of metals inside was evidenced. Enlarged cells with multiple defects in cytoskeletal organisation, caused by individual and aggregated internalised particles in perinuclear areas, confirmed the cytotoxic effect of these metals.

Phase-contrast and Laser Scan Confocal microscope observations of PBMC did not provide a statistically significant quantitative evaluation. However, no diagnostic response, but only the proof of metal particles internalization, was expected.

## Conclusions

In conclusion, to achieve an improvement in clinical practice, a PT confirmed by LTT could be introduced as standard procedure. This would allow the identification of subjects who are likely to develop implant-related hypersensitivity reactions. At the same time, it would avoid the development of allergies from joint implantation, and reveal any reactions due to implant compounds.

## Abbreviations

Co: Cobalt powder
Cr: Chromium powder
CrCl_3_: Chromium (III) chloride
CRP: C-reactive protein
ESR: Erythrocyte sedimentation rate
KSS: Knee society score
LSCM: Laser scan confocal microscopy
LTT: Lymphocyte transformation test
MHC II: Class II major histocompatibility complex
Mo: Molybdenum nanopowder
Ni: Nickel nanopowder
NiCl_2_: Nickel (II) chloride
PBMC: Peripheral blood mononuclear cells
PE: PolyEthylene
PHA: Phytohemagglutinin
PT: Patch test
pTKA: Primary total knee arthroplasty
ROM: Range of motion
SI: Stimulation index
Ti: Titanium powder
TKA: Total knee arthroplasty
WOMAC: Western Ontario and McMaster Universities Osteoarthritis Index
